# Pancreaticobronchial Fistula Diagnosed by MDCT

**DOI:** 10.5334/jbr-btr.890

**Published:** 2015-12-30

**Authors:** G. Verlynde, A. Rezazadeh Azar, Ph. Maldague, O. Van Cutsem

**Affiliations:** 1 Department of Radiology, Clinique St-Luc, Bouge (Namur), Belgium; 2 Department of Gastroenterology, Clinique St-Luc, Bouge (Namur), Belgium; 3 Department of Pneumology, Clinique St-Luc, Bouge (Namur), Belgium

**Keywords:** pseudocyst, pancreaticobronchial fistula, computed tomography

## Abstract

Pancreatic duct disruption is a serious complication of acute or chronic pancreatitis. These ruptures may cause collections of pancreatic secretion leading to ascites but also to pleural or mediastinal effusions. Rupture into the bronchial tree, resulting in a pancreaticobronchial fistula, is also possible, but it is a rare complication. It should be considered if a patient with pancreatitis develops respiratory symptoms and requires cross-sectional imaging to identify pancreaticobronchial fistulae.

## Case Report

A 52-year-old man, referred to us for coughing with sputum and dyspnea, was hospitalized because of septic shock following bilateral pneumonia. Two months previously, he had been hospitalized for a pancreatic pseudocyst during an acute phase of a head pancreatitis. Computed tomography (CT) at that time showed extensive collection around the liver, with a communication with the pancreas. The endoscopic retrograde cholangiopancreatography (ERCP) revealed a large fistula from the Wirsung to this pseudocyst, and a sphincterotomy was realized with the placement of a 7Fr 5-cm prosthesis. During follow-up, about 3 weeks after the patient’s hospitalization because of pneumonia, a new increase in the biological inflammatory syndrome was noticed.

**Figure 1:**
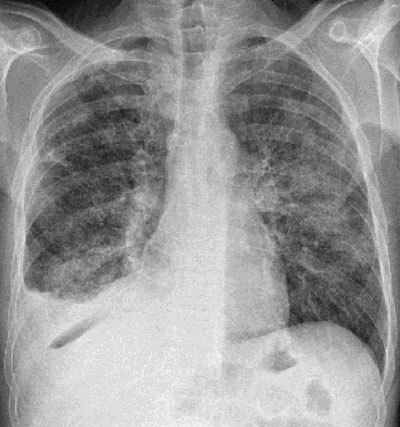
Chest X-ray showing parenchymatous condensation in the middle lobula and a right pleural effusion. A small unusual aeric image is shown under the right section of the diaphragm.

A control radiography showed a persistent parenchymatous condensation in the middle lobula, as well as a right pleural effusion. A small, unusual aeric crescent-shaped picture was seen under the right section of the diaphragm, suggesting a pneumoperitonea (Figure 1).

**Figure 2:**
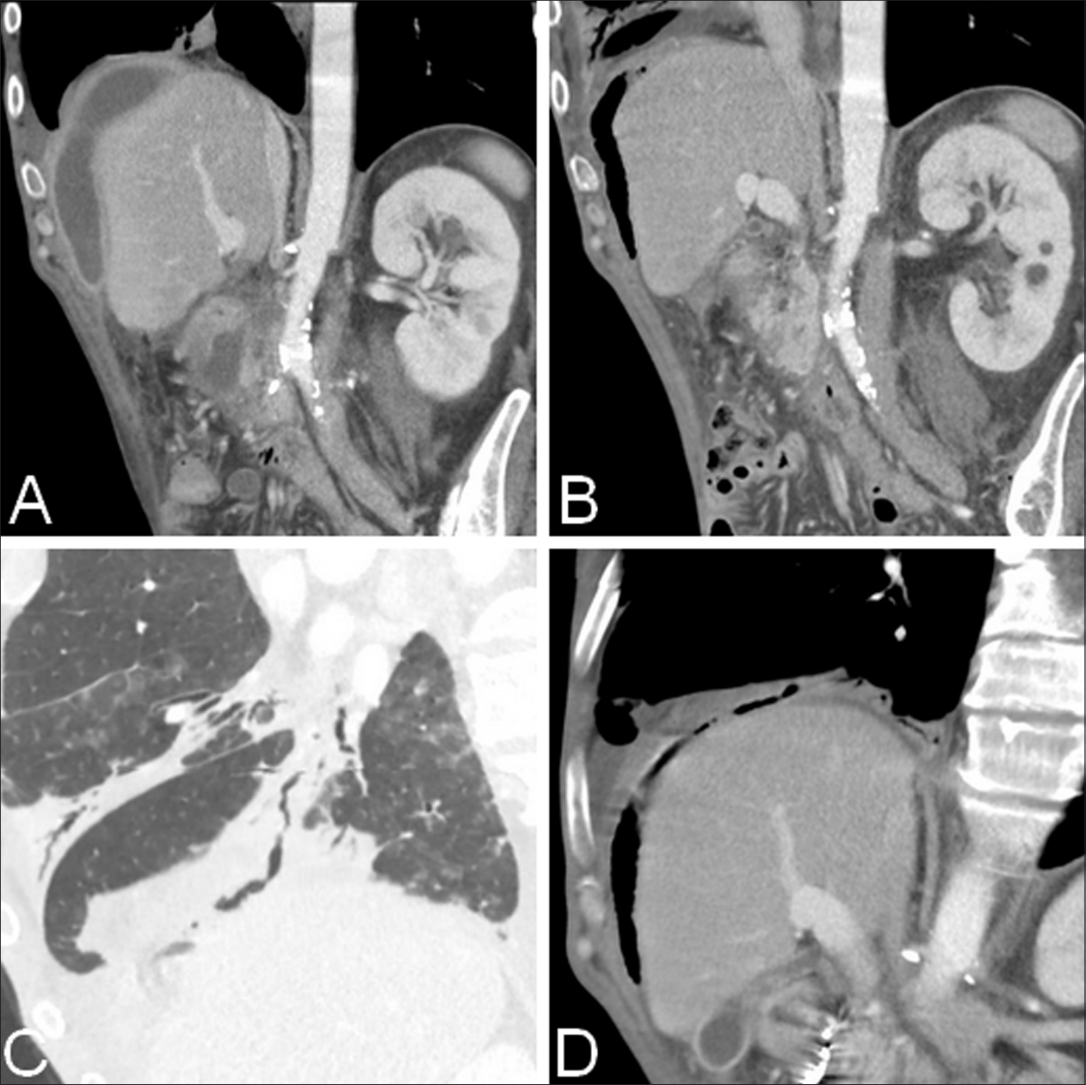
(A) CT showing a pseudocyst underneath the right portion of diaphragm, above the liver. (B) The last exam reveals a significant decrease and apparition of gas in the pseudocyst, suggestive of spontaneous fistula. (C, D) Coronal oblique MPR views respectively in the pulmonary and abdominal windows showing a distal bronchi communicating by a fistulous way to the pseudocyst through the diaphragm.

**Figure 3:**
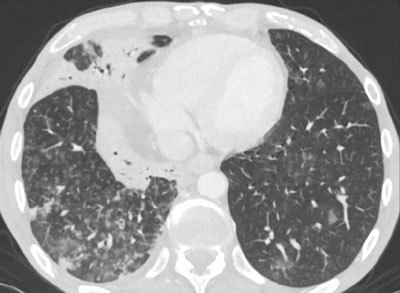
Multiples areas of centrilobular nodules with a linear branching (tree-in-bud pattern) in the right inferior lobula and condensation with air bronchogram in the middle lobula.

CT revealed that the size of the pseudocyst had decreased significantly, and a large amount of gas was observed inside the cyst, suggestive of spontaneous fistula. A distal bronchi communicating by a fistulous way to the pseudocyst was visualized (Figure 2). Multiples areas of centrilobular nodules with a linear branching (tree-in-bud pattern) in the right inferior lobula and a condensation with air bronchogram in the middle lobula were also noticed (Figure 3). Further, the 7Fr 5-cm prosthesis placed in January had fallen in the abdomen. Based on multiple detector computed tomography (MDCT) findings, the diagnosis of pancreaticobronchial fistula was suggested and confirmed by analysis of bronchial expectorations that showed raised lipasis and amylasis.

Conservative treatment by somatostatin was instaured, and the pancreatic duct was stented with a new 10Fr 5-cm stent. The evolution was favorable with improvement on the 1-week follow-up CT scan.

## Discussion

Pancreatic pseudocysts are a common and well-known complication of acute or chronic pancreatitis.

These are collections of pancreatic secretions resulting from a major pancreatic duct disruption. Their most common site is the omental bursa. Sometimes they can rupture into the abdominal cavity, resulting in pancreatic ascites, or reach the thorax, developing either by direct passage through a natural diaphragmatic hiatus (esophageal or aortic) or by fistulation through the dome of the diaphragm. The consequences are bronchopleural fistula or, least commonly, pancreaticopericardial or pancreaticobronchial fistula. A pancreaticobronchial fistula should be considered if a patient with pancreatitis develops cough with sputum, dyspnea, or severe respiratory distress [1,2]. In fact, although the pancreatic enzymes present in the bronchial tree are not activated, they are irritating for the bronchial mucosa and thus promote tracheobronchitis or pneumonia [3].

The diagnosis of the pancreaticobronchial fistula usually relies on imaging. It is suggested by the findings of several imaging modalities. For example, if it is associated with a pancreaticopleural fistula, the chest X-ray may demonstrate a hydropneumothorax in a patient with pancreatitis [4]. ERCP can reveal such a fistula [5]. However, in the majority of the cases, the diagnosis is made by using the contrast MDCT. The findings include pancreatic duct disruption resulting in pseudocyst containing some gas and fistulous tracts to the distal bronchi. At the thoracic level, we observe a tree-in-bud pattern. It was initially described in patients with endobronchial tuberculosis, but it can also be seen with many pulmonary diseases with bronchogenic dissemination. In our case, it results from inflammatory exudate in the bronchiole. The presence of raised amylase and lipase in the sputum are pathognomonic, and it confirms the diagnosis of pancreaticobronchial fistula [6].

Some aspects of the management of a pancreatic pseudocyst may be applicable to the treatment of pancreaticobronchial fistula to control the source of the secretion. The medical management consists of administrating the synthetic somatostatin analog octreotide. It acts by strongly decreasing the pancreatic exocrine secretion, thus decreasing the fistula output. This is combined with other measures such as nasojejunal tube feeding and/or chest drains. Treatment with ERCP and stenting aims to decompress the ductal system with measures such as sphincterotomy, and to bridge the ductal disruption. The stents range from 5F to 7F size and are used for a duration varying from 4 to 12 weeks. Surgery is necessary in patients not responding to conservative and medical management or endoscopic stenting, and in patients with recurrence of symptoms. The most common surgical procedure consists of partial pancreatectomy of the diseased segment and pancreatojejunostomy [7]. It has been demonstrated that prolonged periods of medical therapy tend to delay further the resolution of the fistula as compared with patients who undergo definitive operative intervention early in the course of treatment [8].

Although rare, pancreaticobronchial fistulae should be sought in any patient with cough and/or dyspnea during the episode of acute pancreatitis. MDCT is the best imaging modality to detect the pancreaticobronchial fistula.

## Competing Interests

The authors declare that they have no competing interests.
